# Bacterial composition and colony structure of the lower respiratory tract in infants and children with recurrent wheezing: a case–control study

**DOI:** 10.1186/s13052-022-01279-6

**Published:** 2022-07-19

**Authors:** Jiawei Yao, Tao Ai, Wanmin Xia, Yinghong Fan, Cheng Xie, Lei Zhang

**Affiliations:** Division of Pediatric Pulmonology, Chengdu Women’s and Children’s Central Hospital, School of Medicine, University of Electronic Science and Technology of China, 1617 Riyue Avenue, Qingyang District, Chengdu, CN 610091 Sichuan China

**Keywords:** Microbiome, Bronchoalveolar lavage, Children, Recurrent wheezing

## Abstract

**Background:**

The bacterial load of the human lower respiratory tract is at least several times lower than that of the other parts of the body. This study aimed to identify the bacterial composition and colony structure of the lower respiratory tract in infants and children with recurrent wheezing compared with those of children with a bronchial foreign body and clarify whether the length of wheezing in infants can contribute to differences in the lower respiratory tract’s bacterial colony structure.

**Methods:**

We collected specimens of alveolar lavage fluid from 48 infants and children who underwent fiberoptic bronchoscopy and were divided into groups: A1 (multiple wheezing: wheezing more than three times in < 1 month), A2 (persistent wheezing: > 1 month), and B (bronchial foreign body; control group). We analyzed the bacterial community structure of alveolar lavage fluid using high-throughput sequencing. The richness and diversity of the microbial communities were assessed by α and β diversity analyses.

**Results:**

A total of 6,644 operational taxonomic units (OTUs) were obtained based on the Illumina Nova sequencing platform and clustered according to those that met the 97% identity threshold, followed by species annotation of the OTU sequences. In the annotation results, 2,608 (39.25%) OTUs were annotated at the genus level. At the genus level, *Sphingomonas* and *Phyllobacterium* were significantly higher in group A1 than in group B. There were significantly more *Phyllobacterium* in group A2 than in group B. *Prevotella*, *Neisseria*, and *Haemophilus* were higher in group B than in groups A1 and A2. The differences in the between-group α and β diversity analyses were statistically significant. The microbial diversity in groups A1 and A2 was significantly less than that in group B, but there was no statistical difference in bacterial community diversity between groups A1 and A2.

**Conclusion:**

Recurrent wheezing in infants and children is more likely due to alterations in the overall bacterial microecology and disruption of host respiration and immune homeostasis than the effects of a single bacterium.

**Supplementary Information:**

The online version contains supplementary material available at 10.1186/s13052-022-01279-6.

## Background

Wheezing is a common respiratory symptom in children, especially in infants and young children, and about one-third of children will have at least one episode of wheezing by the age of 3 years, and 1–2% of children are hospitalized for severe respiratory symptoms [1]. Wheezing resolves with age in some infants and children, while others develop persistent wheezing or asthma [2]. Recurrent wheezing in infants and children is often attributed to airway injury, inflammation, and immune responses due to infection, allergy, and environmental factors. However, few reports have shown whether bacterial flora disorders in the lower respiratory tract are associated with recurrent wheezing in infants and children. The human lower respiratory tract’s bacterial load is at least several times lower than that of the other parts of the body and may be highly plastic and susceptible to external forces [3, 4].

The earliest description of symbiotic microorganisms in the body can be traced back to the study of *Enterobacter* in the digestive tract of infants by Escherich more than a century ago [5]. Over the decades, the microbiome’s importance has gradually gained recognition, from early descriptions of gut bacteria in response to diet to the detailed mechanisms of host-microbe interactions found in a large body of work documented in recent years, including pathways affecting systemic immunity, cardiovascular health, and neurological development [6–9]. However, research and understanding of the bacterial community structure of the lung remain scarce as it was long considered a sterile environment, except when infected by respiratory pathogenic microbes. This deeply-rooted notion led to the removal of lungs from the sampling list in the first round of the Human Microbiome Project (HMP) [10]. The second round of the HMP found viruses and fungi in the lung in addition to bacteria, but the health and disease effects of each microorganism are still not well understood [11–14].

In recent years, recurrent wheezing in infants and preschoolers has become very common. The increase in emergency department visits and hospitalization has societal as well as personal effects [15]. The prognosis of recurrent wheezing in infants and children varies, and the onset and duration of wheezing may sometimes be inevitably associated with asthma [16, 17], which has been proven to be caused by impaired lung function due to bacterial infection. *Streptococcus pneumoniae*, *Moraxella catarrhalis,* and *Haemophilus influenzae* are the microbiota primarily associated with the exacerbation of asthma [18, 19]. However, it remains unclear whether prolonged, persistent wheezing versus multiple wheezing episodes cause different changes in the lower respiratory tract’s colony structure.

The ideal method of specimen collection is to collect lung tissue samples by isolating microbial deoxyribonucleic acid (DNA) from host DNA, but this method cannot be routinely applied to every disease. Although sputum specimens are easy to collect, contact with the oral cavity and upper respiratory tract is unavoidable during the collection process. In contrast, bronchoalveolar lavage fluid is immune to upper airway flora contamination and closely mimics the lower airway’s microbiome but cannot wholly sample the lung’s deepest airways. Bronchial foreign bodies are a typical pediatric emergency, commonly affecting infants and children aged six months to three years. Most children with bronchial foreign bodies have a short duration of illness, and mechanical damage to the airway should not adjust the microbiota’s composition over this short period. Therefore, we chose the results of alveolar lavage fluid from children with bronchial foreign bodies instead of lower airway bacterial colonization from healthy infants and children as a control.

Because persistent and repeated wheezing may cause changes in the colony structure of lower respiratory tract microorganisms, the differences in the genera represented by the lower respiratory tract microbiota in infants and children with recurrent wheezing should be elucidated to provide a basis for future insights into the critical role of lower respiratory tract bacteria in the development of recurrent wheezing in infants and children.

Therefore, the first aim of this study was to describe the composition and diversity of bacterial colonies in the lower respiratory tract of children with recurrent wheezing. The second aim was to determine whether persistent wheezing affects infants' and children's microbial colony structure and diversity. The third objective was to identify the dominant bacterial genera in infants and children with recurrent wheezing, provide new ideas for future insights into the role of bacterial genera in regulating airway immunity, and provide new directions for the prevention of diseases that cause recurrent wheezing.

## Materials and methods

### Participants and research design

To analyze the relevant bacterial communities, 48 specimens were collected from the alveolar lavage fluid of children with bronchial foreign bodies or recurrent wheezing admitted to Chengdu Women’s and Children’s Center Hospital. The participants were divided into groups A1 (multiple wheezing: repeated wheezing for a short duration each time; *n* = 13), A2 (persistent wheezing: wheezing for more than one month without previous wheezing; *n* = 16), and B (bronchial foreign body: control group; *n* = 19). The criteria for enrolling children in the wheezing group were (1) age 6–36 months; (2) previous wheezing ≥ three times, wheezing duration < 1 month, or wheezing duration > 1 month; (3) bronchoscopy and alveolar lavage; and (4) no history of antibiotic use for 1 week, no heart disease, tuberculosis, bronchial foreign body, bronchopulmonary dysplasia, abnormal immune function, or abnormal imaging tests. Children in group B had a history of foreign body choking and coughing within the past 3 days, and a foreign body was visible on bronchoscopy.

### Sample collections and molecular analyses

#### Pulmonary function tests

Infant tidal pulmonary function (tidal volume, peak time ratio, peak volume ratio, respiratory ratio), fractional exhaled nitric oxide (Fe–NO), and routine blood tests were completed within 24 h after admission.

#### Bronchoscopy

The guardians of all children signed informed consent forms. Preoperative routine examination and other related tests were performed, and the participants fasted from food and water for 4–6 h. Bronchoscopy was performed with each participant in the supine position under intravenous compound anesthesia with transglottic mask ventilation. The appropriate fiberoptic bronchoscope (OLYMPUS, Tokyo, Japan) was selected according to the child’s age and weight. The bronchoscope was inserted via laryngeal mask airway (LMA) and the child’s epiglottis, tracheal ramus, and each lobe and segment of the bronchus were assessed for lesions.

#### Extraction of alveolar lavage fluid DNA

The DNA in the sample was extracted by the sodium dodecyl sulfate method, and the concentration and purity of the DNA were detected by agarose gel electrophoresis and diluted. Using the diluted genomic DNA as a template, we used foreign objects with Barcode, selected the sequencing area, and used High-Fidelity Polymerase Chain reaction (PCR) Master Mix with GC Buffer (New England Biolabs Phusion®, US) and high-fidelity enzymes for PCR. The amplified regions included the Archaea 16S V4-V5/Archaea 16S V8, 16S V3-V4/16S V4-V5/16SV5-V7; 18S V9, and ITS2 regions.

#### Purification of PCR products, library construction, and up-sequencing

The PCR products were electrophoresed using agarose gel; the quantified PCR products were purified using magnetic beads. Using enzyme labeling quantification, equal amounts of PCR products were mixed according to their concentrations, and PCR products were detected by agarose gel electrophoresis. The products were then recovered for the target bands using the gel recovery kit (Qiagen, Germany). The library was constructed using a DNA PCR-free sample preparation kit (TruSeq®, USA). Qubit and quantitative PCR were used to quantify the constructed library, which was then sequenced using NovaSeq 6000 (Novogene Co., Ltd., Beijing, China).

### Data processing

The sparse algorithm was used to cluster the valid tag sequences into OTUs, which met the 97% identity threshold by default, for all samples. Species annotation of OTU sequences was performed using the Mothur method with the small subunit ribosomal ribonucleic acid (rRNA) database of SILVA138 (with a threshold value of 0.8–1) to obtain taxonomic information and count each sequence at each taxonomic level (kingdom, phylum, class, order, family). The community composition of each sample was counted at each taxonomic level (kingdom, phylum, class, order, family, and genus). The phylogenetic relationships of all OTUs were obtained by rapid multiple sequence matching using MUSCLE software. Finally, the least amount of data in the samples were used as the criterion for homogenization, and the subsequent α and β diversity analyses were based on the homogenized data.

### Statistical analysis and visualization

We drew a light curve to estimate whether the sample’s current sequencing depth could reveal the microbial community’s diversity [20]. QIME software was used to calculate the α diversity index, and differences between the α diversity indices were checked using rank-sum tests [21]. β diversity analysis can detect the similarity of bacterial colony structure between different groups through UniFrac distance, and the non-metric multidimensional scaling (NMDS) method was used for visualization [22–24]. The contribution of the differences between the two groups for each species was quantified using the similarity percentage method (SIMPER) [25, 26]. UPGMA (Unweighted Pair-group Method with Arithmetic Mean) is a more commonly used method for cluster analysis; it can be used to study the similarity between different samples and to perform cluster analysis on samples. In addition, linear discriminant analysis of effect size (LEfSe) was used to screen taxa with rich differences between groups and discover biomarkers [27]. Sequence data analysis and data curation were performed using QIIME, R package (v3.2.0), and IBM SPSS Statistics (v21.0). Independent sample t-tests and chi-square tests were used to analyze the statistically significant differences between continuous and categorical variables, respectively. One-way analysis of variance was used for continuous variables among the three groups (*P < 0.05).

## Results

### Patients’ characteristics

All groups had a higher proportion of male participants, but the between-group sex differences were not statistically significant. The white blood cell count and the percentage of eosinophils in routine blood tests were not significantly different and were within the normal range. We measured fractional exhaled nitric oxide (Fe–NO) and tidal pulmonary function in only 11 cases in group A1 and 13 cases in group A2 (data were missing in the other five cases), in which the mean values of the ratio of tidal expiratory flow to total expiratory time (TPEF/TE) and the ratio of peak expiratory flow to total expiratory flow (VPEF/VE) were lower in group A1 than group A2, suggesting severe obstructive ventilation. However, they were not significantly different between the two groups. The general information regarding the participants is shown in Table 1.

**Table 1:** Participants’ baseline characteristics

Characteristics	A1 (*n* = 13)	A2 (*n* = 16)	B (*n* = 19)	***P***
Age (months)^a^	12.18 ± 4.35	11.92 ± 5.60	15.57 ± 3.502	0.084
Weight (kilogram)^a^	9.25 ± 1.34	9.94 ± 1.01	10.46 ± 1.37	0.066
Gender (male)^b^	10 (76.9%)	10 (62.5%)	12 (63.2%)	0.655
Duration of disease (months)^a^	0.27 ± 0.19	3.46 ± 2.88	0.06 ± 0.04	< 0.05
Blood eosinophils (%)^a^	2.11 ± 2.11	2.32 ± 1.70	2.28 ± 1.26	0.992
White blood cell count (× 10^6^/g) ^a^	9.29 ± 3.61	11.24 ± 4.10	9.24 ± 2.28	0.245
Fe–NO (ppb)^c^	10.64 ± 3.78	9.69 ± 3.61	-	0.539
Tidal volume (mL/kg)^c^	8.58 ± 1.45	7.86 ± 1.56	-	0.264
VPEF/VE (%)^c^	20.23 ± 2.58	22.77 ± 5.61	-	0.181
TPEF/TE (%)^c^	17.16 ± 3.96	20.57 ± 7.14	-	0.174
Respiratory rate (times/min)^c^	32.09 ± 8.17	30.77 ± 7.04	-	0.674

^a^The values shown for continuous variables are means ± standard deviations. One-way analysis of variance

^b^The categorical variables show the number of samples, and the percentage is shown in parentheses

^c^The values shown for continuous variables are means ± standard deviations. Independent sample t-tests

### Bacterial abundance and Distribution

The PCR-free library was sequenced by paired-end sequencing using the NovaSeq 6000 (Illumina, USA). By splicing the reads, an average of 87,349 tags were sequenced per sample, and 81,331 valid data points were obtained after quality control, resulting in 61,798 valid data points in quality control and a quality control efficiency of 71.37%. The sequences were clustered into OTUs that met the 97% identity threshold, and 6,644 OTUs were identified. Between 182 and 1326 OTUs (mean = 565) were identified per sample in group A, and 282–1334 OTUs (mean = 580) were identified per sample in group B.

**Fig. 1:**
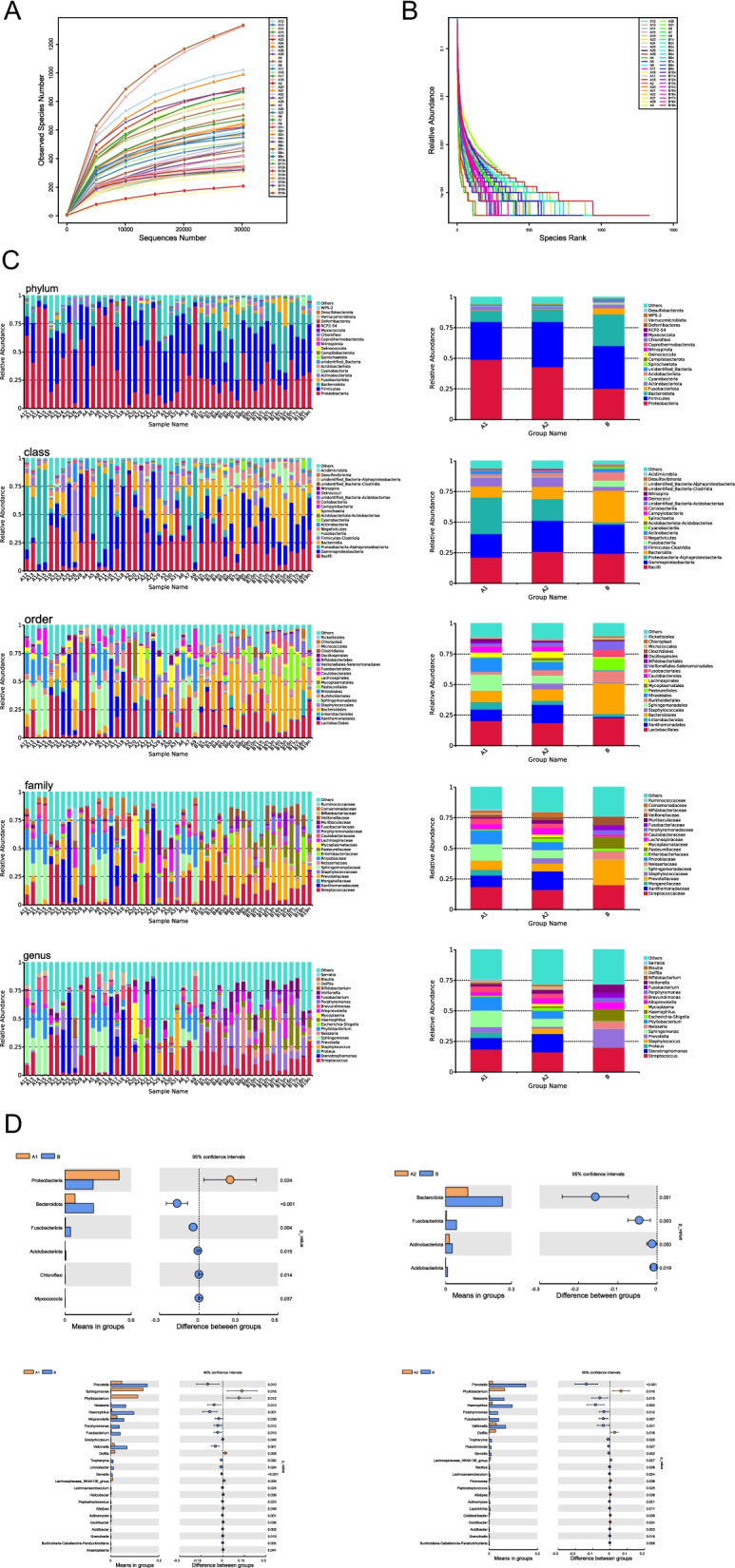
Multiple rarefaction curves for all the alveolar lavage fluid samples. Multiple rarefaction curves of the (**A**) richness and (**B**) rank abundance (**B**). **C** Columnar cumulative chart of relative abundance of the top 20 species with the greatest abundance at the phylum, class, order, family, and genus levels. **D** Distribution of dominant bacteria in groups A1, A2, and B at the phylum and genus levels

The richness sparsity curves for groups A1, A2, and B confirmed that all the samples had sufficient sequencing depth (Fig. 1A), and the rank clustering curves reflected the samples’ uniform distribution and high species richness (Fig. 1B). Based on the annotation results, the 20 most abundant species at the phylum, order, family, and genus levels were selected for each sample or group, and a cumulative bar graph of the species’ relative abundance was generated (Fig. 1C).

A total of 11 phyla, 28 classes, 67 orders, 99 families, and 165 genera were detected in the alveolar lavage fluid specimens, which could be annotated to 70.64% at the phylum level, 68.68% at the class level, 64.77% at the order level, 56.17% at the family level, and 39.25% at the genus level. The major bacterial distributions were characterized based on relative taxonomic abundance (Fig. 1D) as Proteobacteria (A1 = 49.1%, A2 = 43.2%, B = 25.5%), Firmicutes (A1 = 30.9%, A2 = 36.9%, B = 34.9%), Bacteroidota (A1 = 9.1%, A2 = 10.1%, B = 25.9%), and Fusobacteriota (A1 = 0.5%, A2 = 0.4%, B = 5.0%), and at the genus level with *Streptococcus* (A1 = 18.8%, A2 = 16.4%, B = 20.1%) *Stenotrophomonas* (A1 = 9.2%, A2 = 14.9%, B = 0.1%), *Prevotella* (A1 = 4.5%, A2 = 1.4%, B = 15.1%), *Sphingomonas* (A1 = 13.4%, A2 = 6.5%, B = 0.1%), *Staphylococcus* (A1 = 0.8%, A2 = 4.3%, B = 0.1%), *Phyllobacterium* (A1 = 11.2%, A2 = 6.3%, B = 0.0%), *Neisseria* (A1 = 0.1%, A2 = 0.3%, B = 6.1%), *Haemophilus* (A1 = 0.5%, A2 = 1.1%, B = 9.6%), *Brevundimonas* (A1 = 4.6%, A2 = 3.7%, B = 0.5%), *Porphyromonas* (A1 = 0.0%, A2 = 0.0%, B = 3.5%), *Fusobacterium* (A1 = 0.3%, A2 = 0.01%, B = 4.0%), and *Veillonella* (A1 = 1.5%, A2 = 2.9%, B = 6.8%)( Table 2).

**Table 2:** Major bacterial distributions characterized based on relative taxonomic abundance

phyla	**A1**	**A2**	**B**
Proteobacteria	49.1%	43.2%	25.5%
Firmicutes	30.9%	36.9%	34.9%
Bacteroidetes	9.1%	10.1%	25.9%
Fusobacteriota	0.5%	0.4%	5.0%
genus	**A1**	**A2**	**B**
*Streptococcus*	18.8%	16.4%	20.1%
*Stenotrophomonas*	9.2%	14.9%	0.1%
*Prevotella*	4.5%	1.4%	15.1%
*Sphingomonas*	13.4%	6.5%	0.1%
*Staphylococcus*	0.8%	4.3%	0.1%
*Phyllobacterium*	11.2%	6.3%	0.0%
*Neisseria*	0.1%	0.3%	6.1%
*Haemophilus*	0.5%	1.1%	9.6%
*Brevundimonas*	4.6%	3.7%	0.5%
*Porphyromonas*	0.0%	0.0%	3.5%
*Fusobacterium*	0.3%	0.01%	4.0%
*Veillonella*	1.5%	2.9%	6.8%

Genus groups with significant differences at the level of bacterial phylum and genus were assessed by performing t-tests for groups A1, A2, and B. At the phylum level, the number of Bacteroidetes bacteria in groups A1 and A2 were lower than in groupB, and the number of Proteobacteria were higher in groups A1 and A2 than in group B. At the genus level, the numbers of *Sphingomonas* and *Phyllobacterium* in group A1 were higher than those in group B. Furthermore, the number of *Phyllobacterium* in group A2 was higher than that in group B. *Prevotella*, *Neisseria*, and *Haemophilus* were more in group B than in groups A1 and A2.

### Bacterial diversity and colony structure analysis

**Fig. 2:**
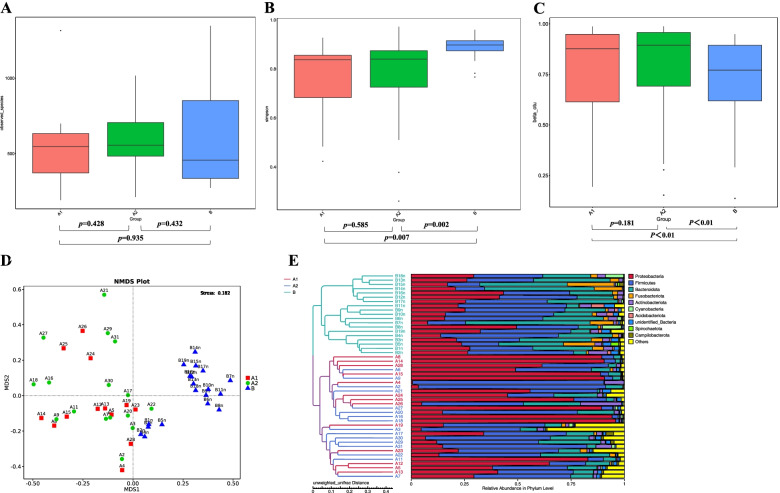
Bacterial diversity in alveolar lavage fluid samples of children in groups A1, A2, and B. **A** The number of observed species. **B** α diversity shown by Simpson's Diversity Index. **C** β diversity expressed as the Bray–Curtis distance in the alveolar lavage fluid samples. **D** NMDS based on the Bray–Curtis distances at the OTU level which met the 97% identity threshold. **E** UPGMA clustering of the microbiome classification maps in the three groups using the UniFrac distance showed relatively clear separation between groups

There was no significant difference in the number of OTUs directly observed among the three groups (Fig. 2A). The α diversity in group B was significantly different from the Simpson’s Diversity Index of groups A1 and A2, and the diversity and evenness of group B were higher than those of groups A1 and A2 (Fig. 2B). In the analysis of β-diversity, we found that the Bray–Curtis distance of the bacterial community in group B was different from that in groups A1 and A2. There was no statistically significant difference in the Bray–Curtis distance between group A1 and group A2 (Fig. 2C). NMDS plots showed similar distances between groups A1 and A2 and were distant from group B, suggesting that the colony structures of groups A1 and A2 are highly similar and differ from those of group B (Fig. 2D). The specific information regarding this is shown in Additional file 1: Appendix 1. UPGMA clustering of microbiome classification profiles in all groups with UniFrac distance distances showed relatively significant difference between the control and wheezing groups (Fig. 2E).

### Differential microbiota composition

The species abundance data between groups were analyzed using LEfSe analysis to detect the differential species between groups by rank-sum test and to assess the effect size of differential species by linear discriminant analysis (LDA). Histograms of the distribution of LDA values of the differential species and evolutionary branching of the differential species were plotted. We found 47 biomarkers with an LDA score > 4, including *Haemophilus*, *Neisseria*, *Prevotella*, *Sphingomonadales*, *Sphingomonas*, *Delftia*, and *Veillonella*.

**Fig. 3:**
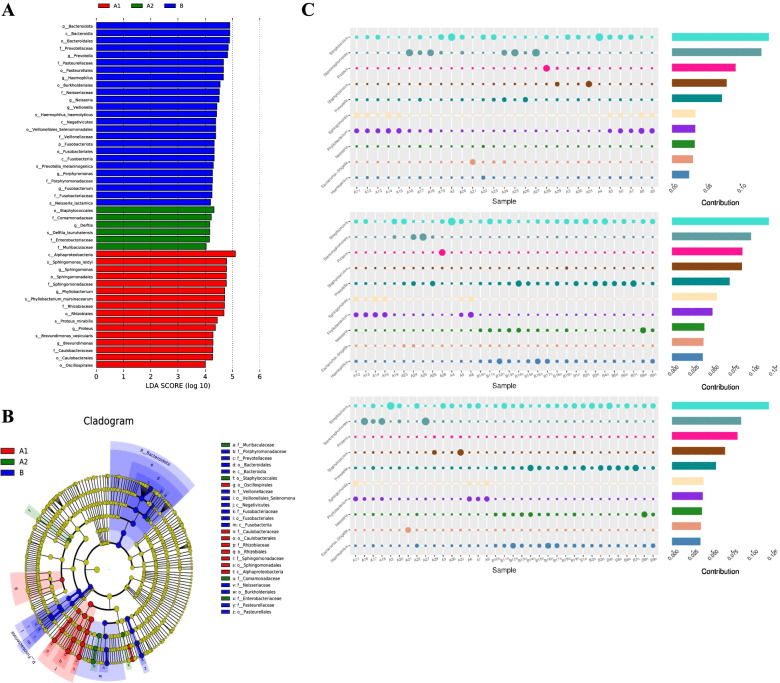
The linear discriminant analysis score (**A**) and taxonomy tree (**B**) of the significant between-group differences. The top 10 genera and their abundances in the difference contribution between groups A1, A2, and B

These differentially rich taxa can be considered potential biomarkers (Fig. 3A; see S1 for details) and are shown in LEfSe plots in order of phylum to genus and group importance (the microbial population with similar coloring as the group is more important in that group; Fig. 3B). The contribution of the top ten species in abundance to the variability between each group was quantified by SIMPER (bubble size represents the relative abundance of the species, and contribution is the species’ contribution to the variability between the two groups; Fig. 3C). Anosim’s test showed that the differences between groups B, A1, and A2 were greater than those within the groups (Table 3).

**Table 3:** Analysis of the differences between Anosim groups

**Group**	***R*-value**	***P*-value**
**A1-B**	**0.6604**	**0.001**
**A2-B**	**0.5916**	**0.001**

An *R*-value between (-1, 1) and > 0 indicates significant between-group differences. An *R*-value < 0 indicates that the within-group difference was more significant than the between-group difference

## Discussion

Humans have evolved symbiotically with microorganisms, and host-microbial interactions are gradually being elucidated, with bacterial colonization of the human host impacting a variety of diseases [28]. Microbial colonization can be detected in the naso- and oropharynx of newborns within the first 5 min after birth, indicating that colonization of the upper respiratory tract has begun [29]. Initially, the immune system of newborns will adjust so that the microbiota can colonize the respiratory tract smoothly. The stable symbiotic microbiota will gradually promote the improvement of the neonatal immune system, and the establishment of this microbiota gives the body its ability to fight pathogenic bacteria. The resident microbiota and the infant’s immune system are restricted in many ways and regulated by metabolites and microorganisms. Immunoglobulin A, present in breast milk, can help establish a stable host-microbiota relationship [30]. Bacteria, viruses, and fungi are the primary pathogens responsible for human respiratory infections. However, in 30% of respiratory cases, pathogens remain undetected by conventional methods [31].

Data released in 2017 suggest that the bacterial microbiota differs slightly between bronchial lavage fluid and endotracheal samples [32]. Our analysis of alveolar lavage fluid microbiomes from 48 children showed that the lower respiratory flora of those in the wheezing group (showing both recurrent and persistent wheezing) comprised mainly the phylum Proteobacteria, followed by the thick-walled Firmicutes and the Bacteroidota.

In contrast, the alveolar lavage fluid specimens from children in the foreign body group (control group) were mainly composed of the phylum Firmicutes, followed by Proteobacteria and Bacteroidota. There is a relative lack of data on children’s lower respiratory flora, and studies of oropharyngeal microflora have confirmed that the phyla mentioned above were found to be dominant in both asthmatic and healthy populations [33, 34]. A neonatal-based study showed that *H. influenzae*, *M. catarrhalis*, and *S. pneumoniae* were associated with wheezing outcomes within the first 5 years of life. Although there is no direct evidence for the cellular impact of these bacterial fixations, these associations may be consistent with significant neutrophil elevation in children with severe recurrent wheezing [18, 35].

We compared the wheezing and foreign body groups in terms of microbial genera and observed abundant *Streptococcus* spp. in each group. The numbers of bacteria of genera *Sphingomonas* and *Phyllobacterium* were significantly higher in the recurrent wheezing group than in the control group; *Phyllobacterium* spp. were also higher in the prolonged wheezing group than in the control group, and *Prevotella* spp., *Neisseria* spp., and *Haemophilus* spp. were higher in the control group than in the wheezing groups.

The low proportions of *Moraxella* spp., *Staphylococcus* spp., and *Haemophilus* spp. in the wheezing group were different from the increased proportions of *Staphylococcus* spp., *Moraxella* spp., and *Haemophilus* spp., which have been previously reported to increase the risk of chronic wheezing in children [35–40], and require further investigation. The high proportion of *Phyllobacterium* spp. has not been reported to be associated with wheezing in infants and children. However, Wen et al. found a higher enrichment of *Phyllobacterium foliaris* among oropharyngeal microorganisms in children with the influenza A virus; the exact mechanism remains unclear [41].

Reduced numbers of *Prevotella* spp. were found in both wheezing groups. *Prevotella* spp. is a common commensal fixer at mucosal sites and is the dominant respiratory genus [42]. The homeostatic role of *Prevotella* spp. in the healthy lung remains largely unclear. Inflammation and pathology in the chronic obstructive pulmonary disease-like lungs of mice induced by lipopolysaccharide or lipopolysaccharide elastase inhalation were reduced by this genus and mediated the growth of *Pseudomonas* and *Lactobacillus* spp. This suggests that the reduction of *Prevotella* spp. (possibly by creating a microenvironment unsuitable for the growth of the genus, which leads to a decrease in its abundance) in patients with wheezing may not be a pre-disease risk factor.

Some authors have compared the inflammatory properties of *Prevotella* spp. in the lungs of healthy individuals with those of bacilli associated with pulmonary disease in asthmatic patients (*H. influenzae* B, *H. influenzae,* and *M. catarrhalis*). *Prevotella* was found to induce similar levels of CD83, CD86, and CD40 activation marker surface expression, but compared with Proteobacteria, it reduced the levels of interleukin (IL)-12p70, IL-23, and IL-10 cytokines in monocyte-derived dendritic cells. The lower inflammatory capacity of *Prevotella* was also confirmed in mice, where *Prevotella* reduced the production of macrophage inflammatory protein 2-α (IL-8), tumor necrosis factor-a (TNF-a), and thymic stromal lymphopoietin by lung stromal cells, as well as the production of TNF-a by lung immune cells, suggesting that *Prevotella* may be well tolerated in the lungs [43–45].

A high proportion of *Streptococcus* spp. was found in all three groups, and it has been shown that increased abundance of *Moraxella* and *Streptococcus* spp. in nasopharyngeal swabs from infants with capillary bronchitis is associated with recurrent wheezing in children at 3 years of age [46]. This may suggest that infant wheezing is more likely to result from a disruption of overall bacterial microecology and host respiratory and immune homeostasis rather than the influence of a single bacterium. Bacterial detection in the lower airway may be due to an imbalance between constant exposure to upper respiratory microorganisms through micro-inhalation and clearance by endothelial cilia and coughing in the respiratory tract.

The exact reasons for the differences in the dominant genera are unclear and may lead to molecular changes in the mucin glycoproteins during long-term chronic inflammation; mucin glycans are a significant source of nutrients and adhesion sites for bacteria and fungi in diseased airways, and different microorganisms can utilize specific mucins, which influence airway fixation [47]. The physical and chemical factors in the airway (including, oxygen tension, pH, temperature, and mucus) vary with the airway’s anatomical position [48], which, together with irregular medication use in children with multiple and persistent wheezing, may lead to the establishment of new, stable, and complex microflora in the presence of immune dysregulation.

The microbial diversity of the wheezing group was lower than that of the foreign body group, and the diversity of microbial colonies was reduced. The exact mechanism by which bacteria cause wheezing in infants and children remains unclear. The immune balance between the bacteria and host organism may be disrupted. Bacteria and their products (such as endotoxins) can stimulate the production of cytokines in airway epithelial cells. Some cytokines (such as nuclear factor kappa B) can activate the IL-8 gene and then initiate positive feedback regulation to increase the production of neutrophils and inflammatory factors, such as IL-8, IL-6, and TNF-α. The increase in TNF-α production can increase the reactivity of the airway’s smooth muscles, thereby aggravating airway obstruction and causing wheezing [49–51].

Pulmonary function tests can help in judging the severity of diseases with wheezing as a symptom and also in evaluating clinical efficacy and prognosis [52]. The most commonly used lung function test for infants and young children is the tidal breathing lung function test [53]. Both TPEF/TE and VPEF/VE are important parameters reflecting obstructive ventilation disorders, with a normal range of 28–55%. Depending on the degree of obstruction, it is classified as mild obstruction (23–28%), moderate obstruction (15–22%), and severe obstruction (< 15%) [54]. The pulmonary function of the children we observed with wheezing suggested a decrease in severe obstructive ventilation. However, the mean TPEF/TE and VPEF/VE in the multiple wheezing groups were worse than those in the persistent wheezing group, and we speculated that perhaps the persistent wheezing was due to persistent airway spasm caused by some infection, while the more severe pulmonary impairment in the recurrent wheezing group may have caused a greater chance of asthma. However, why the duration of disease differs in patients with similar colony structure is subject to further research. We speculate that this may be related to insufficient and ineffective treatment of infants or children.

The main advantage of our study is that the matched case–control excludes the deviation caused by the influence of age, time, and sex. We also elucidated that the duration of wheezing can affect the structure of the bacterial microbial community. We used alveolar lavage fluid samples obtained via bronchoscopy, which is closer to the proper lower respiratory tract microbial colony composition, and the bronchoscopic foreign body group as the control, which is closer to the community composition of respiratory bacteria in children of the same age.

However, our study had some limitations. We primarily focused on the bacterial group of microorganisms and could not exclude the possibility that viruses and fungi might be involved in recurrent wheezing in children. As in other observational studies, our findings do not necessarily suggest causality. 16S rRNA sequencing allows the annotation between genus- and species-level identification of bacteria but does not provide the resolution of macrogenomic techniques (e.g., birdshot sequencing), especially for closely related species, such as *Streptococcus* spp. and *Prevotella* spp. Specific species may need further discussion.

## Conclusion

Our study showed that the lower respiratory flora of children with wheezing consisted mainly of the phylum Proteobacteria, followed by the phylum Firmicutes and the phylum Bacteroidota, whereas the lower respiratory flora of healthy children may be mainly phylum Firmicutes, followed by Proteobacteria and Bacteroidota. A decrease in the number of *Prevotella* spp might be associated with recurrent wheezing in infants and children. It might be involved in the establishment of homeostasis in the pulmonary environment, the mechanism of this is still unclear. When comparing the diversity of bacterial colony structure in the lower respiratory tract of children with persistent wheezing and those with repeated wheezing, we found that there may not be any particular differences in microbiology between them. Wheezing in infants and children is more likely to be due to disruption of the overall bacterial microecology and host respiratory immune balance rather than the effect of a single bacterium.

Overall, our findings suggest that diagnostic methods for microbiota should be further refined and that longitudinal studies related to animal models, such as the alveolar lavage fluid overall fixation in children with recurrent wheezing, could be refined to explore the role of microbial colonization in children with wheezing. Additional studies are needed to confirm whether changes in overall microbial colonization can reverse the clinical symptoms of recurrent wheezing in infants and children.

## Supplementary Information

**Additional file 1: Table 1. **Alpha diversity indices of all oropharyngeal samples. **Fig. 1.** Multiple rarefaction curves of the richness and Rank Abundance. **Fig. 2 **NMDS based on the Bray-Curtis distances at the OTU level at 97% identity. **Fig. 3 **All significantly different phyla and genera detected by LefSe.

## Data Availability

All data generated or analyzed in this study are included in this published article and its supplementary files.

## Abbreviations

DNA: Deoxyribonucleic acid
Fe–NO: Fractional exhaled nitric oxide
HMP: Human Microbiome Project
IL: Interleukin
LDA: Linear discriminant analysis
LEfSe: Linear discriminant analysis of effect size
NMDS: Non-metric multidimensional scaling
OTU: Operational taxonomic unit
rRNA: Ribosomal ribonucleic acid
TNF-a: Tumor necrosis factor-a
TPEF/TE: The ratio of tidal expiratory flow to total expiratory time
VPEF/VE: The ratio of peak expiratory flow to total expiratory flow
