# Supplementary material for: Bacterial composition and colony structure of the lower respiratory tract in infants and children with recurrent wheezing: a case–control study
Source: Ital J Pediatr. 2022 Jul 19;48:120. doi: 10.1186/s13052-022-01279-6 (PMC9297564; doi:10.1186/s13052-022-01279-6)

**Contents:**

**Table 1| Alpha diversity indices of all oropharyngeal samples**

**FIG 1丨Multiple rarefaction curves of the richness and Rank Abundance**

**FIG 2丨NMDS based on the Bray-Curtis distances at the OTU level at 97% identity**

**FIG 3丨All significantly different phyla and genera detected by LefSe**

**Alpha diversity indices of all oropharyngeal samples**

|  | observed_species | goods_coverage | PD_whole_tree | shannon | simpson | chao1 | ace |
| --- | --- | --- | --- | --- | --- | --- | --- |
| A11 | 499 | 0.996257 | 196.8469 | 3.684013 | 0.790219 | 552.1765 | 589.3008 |
| A12 | 585 | 0.994931 | 203.4484 | 4.413506 | 0.855697 | 699 | 717.549 |
| A13 | 557 | 0.995892 | 160.5508 | 4.935807 | 0.895128 | 648.8795 | 661.1643 |
| A14 | 326 | 0.997018 | 91.64605 | 2.578327 | 0.683177 | 389.5714 | 404.9723 |
| A15 | 331 | 0.996853 | 106.5366 | 2.87252 | 0.767891 | 385.4512 | 421.0462 |
| A16 | 479 | 0.992911 | 70.41971 | 2.168192 | 0.510598 | 700.2718 | 769.2819 |
| A17 | 862 | 0.99142 | 203.1039 | 5.155276 | 0.915955 | 1059.698 | 1089.385 |
| A18 | 403 | 0.994865 | 59.50015 | 2.448007 | 0.626485 | 560.0395 | 561.4821 |
| A19 | 1329 | 0.985026 | 579.0952 | 5.134339 | 0.847409 | 1750.182 | 1763.799 |
| A2 | 210 | 0.99735 | 79.00854 | 1.503328 | 0.378213 | 273.2 | 302.4068 |
| A20 | 631 | 0.992646 | 104.4148 | 3.807161 | 0.830103 | 816.8409 | 854.0856 |
| A21 | 561 | 0.996985 | 65.81551 | 4.774703 | 0.849652 | 594.843 | 615.6801 |
| A22 | 503 | 0.992844 | 258.4041 | 3.748124 | 0.831232 | 816.7838 | 825.9187 |
| A23 | 616 | 0.996124 | 379.8245 | 4.749329 | 0.852719 | 678.2569 | 693.9373 |
| A24 | 643 | 0.994468 | 116.9637 | 4.731066 | 0.837349 | 780.2376 | 785.1232 |
| A25 | 649 | 0.99195 | 74.70733 | 2.795408 | 0.483244 | 892 | 956.1568 |
| A26 | 424 | 0.994832 | 57.61982 | 4.744877 | 0.927253 | 601.7941 | 622.3625 |
| A27 | 486 | 0.992347 | 61.72382 | 1.509679 | 0.260777 | 784.4831 | 887.5034 |
| A28 | 428 | 0.994534 | 94.43221 | 2.857985 | 0.648172 | 636.1538 | 623.3847 |
| A29 | 836 | 0.994269 | 258.0968 | 6.812532 | 0.972224 | 924.5595 | 966.1334 |
| A3 | 792 | 0.99248 | 644.1675 | 5.190097 | 0.883784 | 1005.758 | 1000.086 |
| A30 | 1012 | 0.992016 | 232.7032 | 6.700248 | 0.950223 | 1236.186 | 1199.823 |
| A31 | 623 | 0.995097 | 191.6975 | 4.553361 | 0.856748 | 705.4091 | 734.6526 |
| A4 | 181 | 0.997482 | 55.5964 | 1.420443 | 0.42311 | 260.1667 | 262.0904 |
| A5 | 703 | 0.992016 | 259.4401 | 4.757216 | 0.887151 | 963.5405 | 975.6827 |
| A6 | 534 | 0.99513 | 112.1943 | 4.374541 | 0.860042 | 673.3636 | 677.3728 |
| A7 | 685 | 0.993706 | 275.7827 | 4.764301 | 0.871508 | 837.161 | 858.365 |
| A8 | 372 | 0.995826 | 130.9278 | 2.629954 | 0.708592 | 491.3182 | 502.8125 |
| A9 | 327 | 0.996687 | 86.44138 | 2.790622 | 0.758816 | 418.6667 | 424.9806 |
| B1n | 874 | 0.99301 | 61.63274 | 5.691723 | 0.927156 | 993.1129 | 1043.161 |
| B2n | 824 | 0.992281 | 59.67936 | 5.260302 | 0.871453 | 1040.224 | 1055.267 |
| B3n | 995 | 0.990426 | 68.6921 | 5.649863 | 0.925066 | 1301 | 1244.183 |
| B4n | 468 | 0.994169 | 41.30026 | 3.625433 | 0.767095 | 635.3913 | 674.2963 |
| B5n | 900 | 0.994269 | 64.76663 | 6.166126 | 0.948363 | 1001.211 | 1016.534 |
| B6n | 363 | 0.995594 | 37.3343 | 3.955479 | 0.876676 | 494.0149 | 507.849 |
| B7n | 458 | 0.995031 | 37.2678 | 4.427336 | 0.893985 | 611.0822 | 612.8404 |
| B8n | 344 | 0.995958 | 29.21346 | 3.20759 | 0.782637 | 435.1235 | 477.5535 |
| B9n | 581 | 0.994468 | 45.38497 | 4.814934 | 0.89837 | 710.5421 | 739.7731 |
| B10n | 745 | 0.993971 | 51.72495 | 5.923062 | 0.960143 | 860.993 | 889.6083 |
| B11n | 874 | 0.990989 | 76.76106 | 5.19569 | 0.864213 | 1107.266 | 1157.141 |
| B12n | 352 | 0.997482 | 35.45646 | 4.617362 | 0.911884 | 396.5313 | 420.3591 |
| B13n | 342 | 0.997913 | 27.28871 | 4.769626 | 0.918341 | 381.8571 | 391.2443 |
| B14n | 307 | 0.997979 | 36.15574 | 3.998099 | 0.832919 | 345.125 | 355.2739 |
| B15n | 326 | 0.998045 | 27.81666 | 4.768146 | 0.912064 | 361.6458 | 370.9153 |
| B16n | 353 | 0.997913 | 36.17868 | 4.643636 | 0.907971 | 394.5532 | 395.5561 |
| B17n | 324 | 0.997714 | 29.75341 | 4.213452 | 0.879561 | 384.1538 | 374.3716 |
| B18n | 270 | 0.998045 | 26.7553 | 4.231133 | 0.891165 | 304.22 | 324.5747 |
| B19n | 1344 | 0.986252 | 93.35519 | 6.025708 | 0.897979 | 1700.452 | 1730.918 |


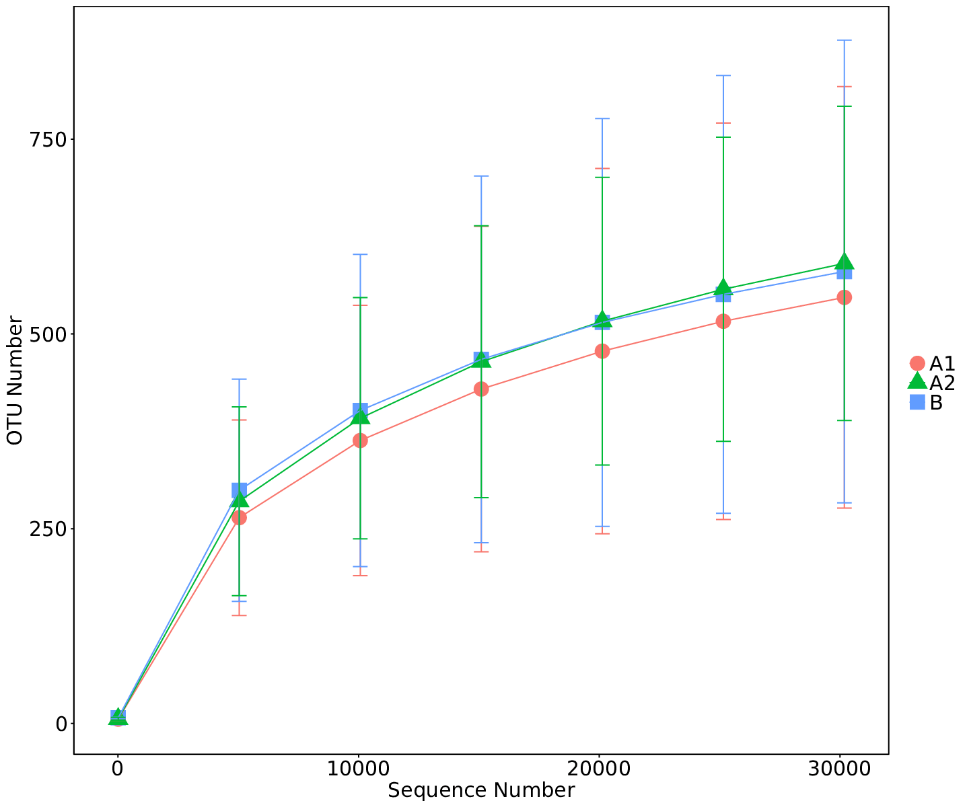

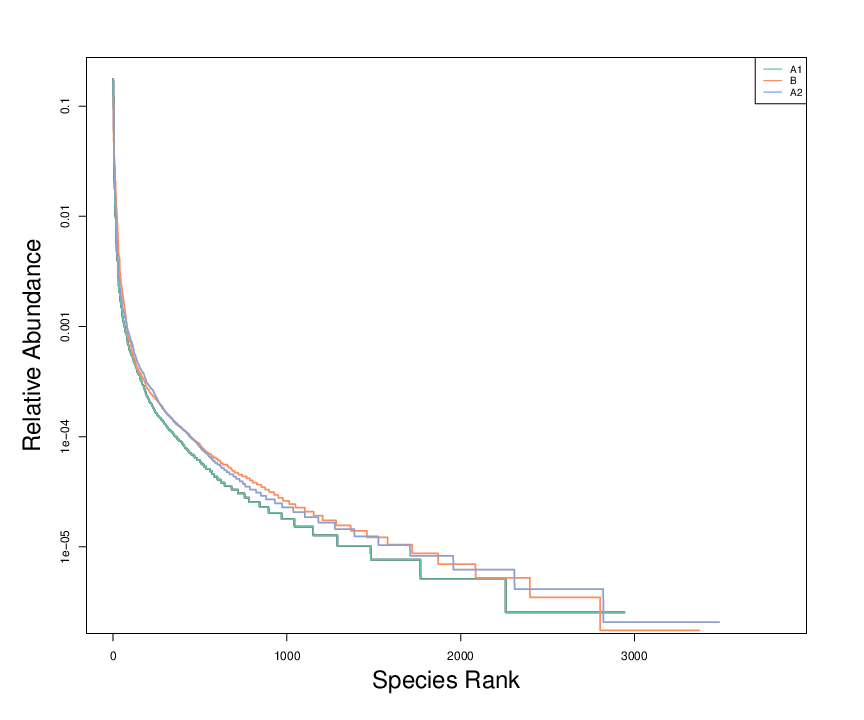
**Multiple rarefaction curves of the richness and Rank Abundance**

**NMDS based on the Bray-Curtis distances at the OTU level at 97% identity**


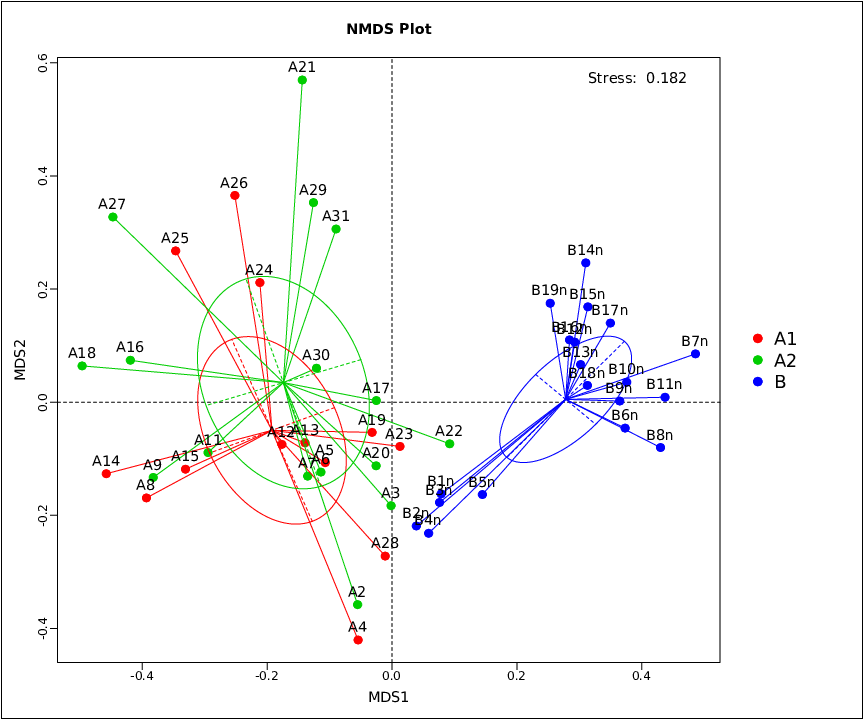


**All significantly different phyla and genera detected by LefSe**
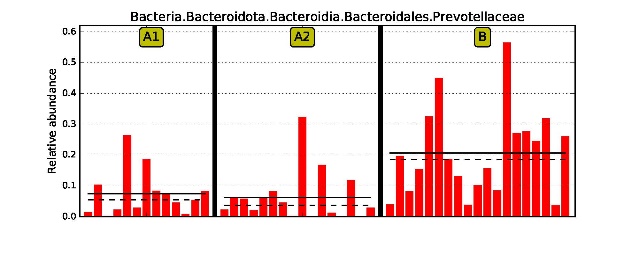

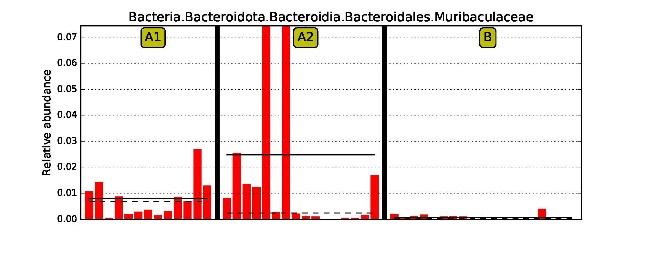

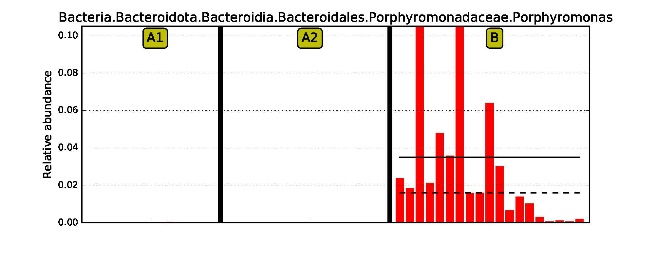


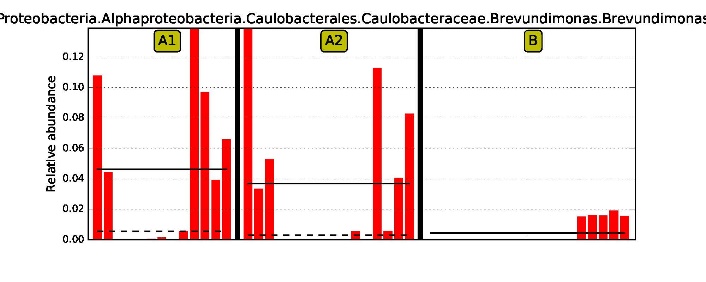

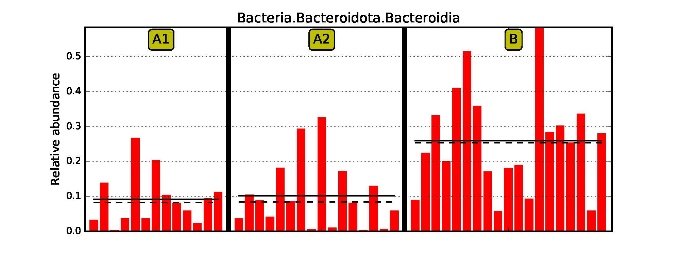

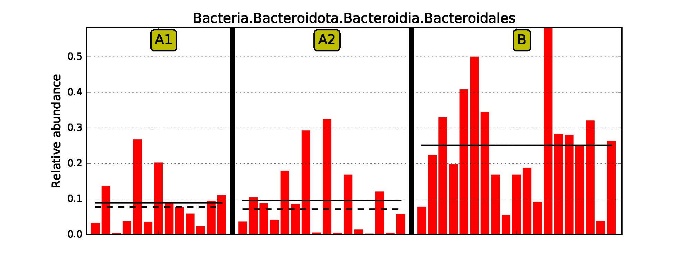

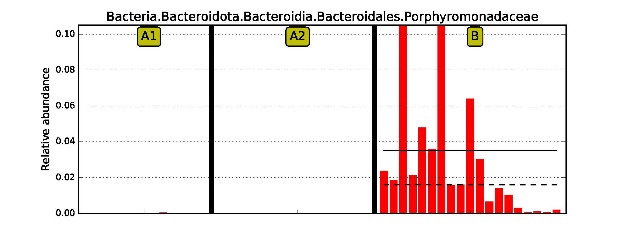


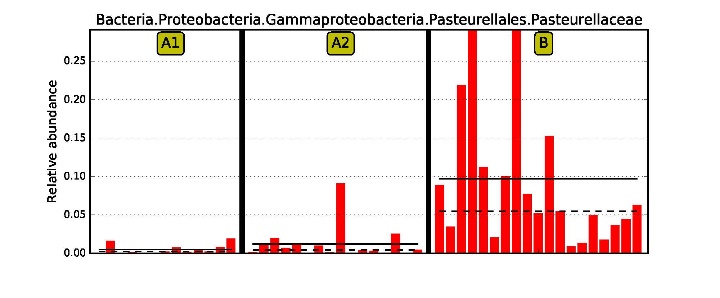

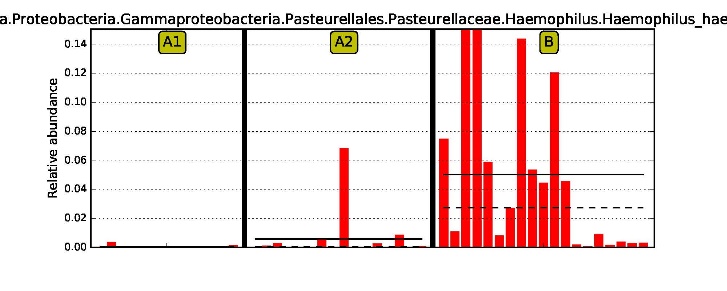

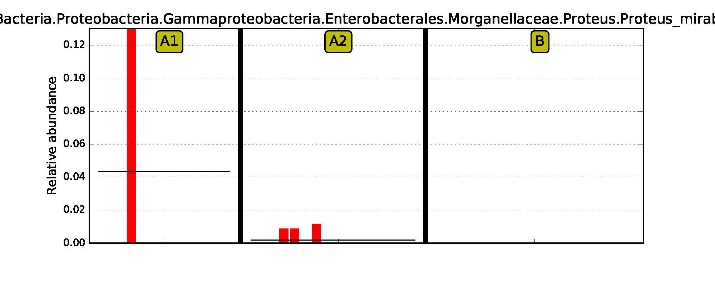

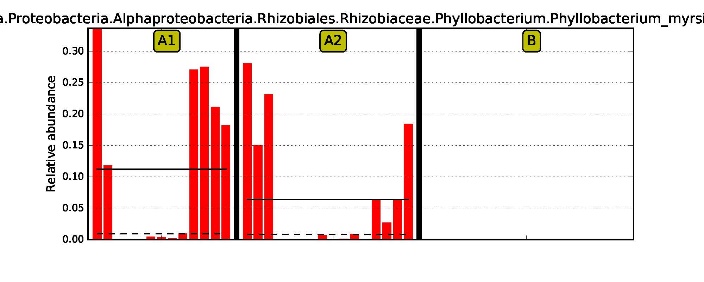

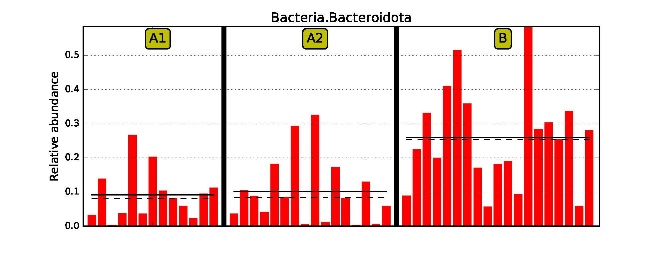


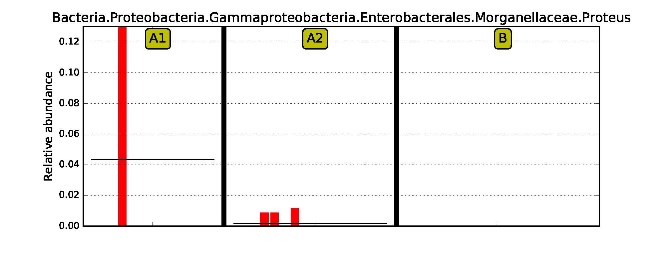

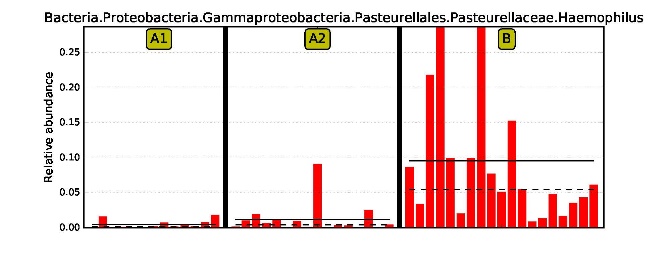


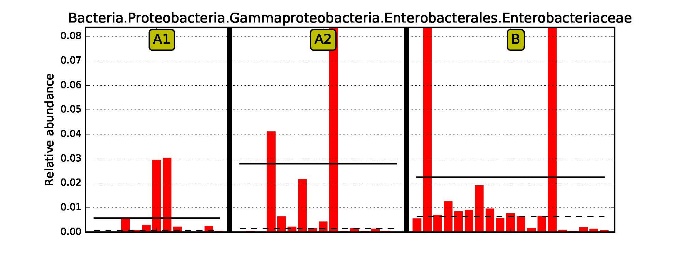


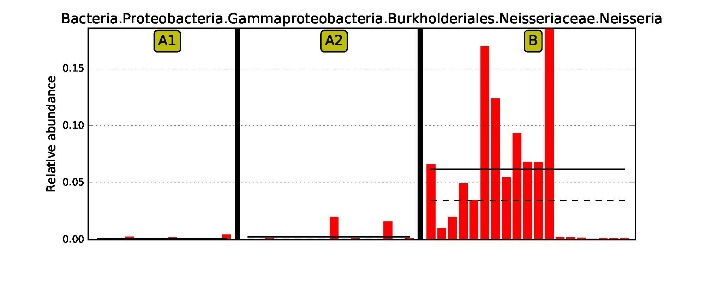

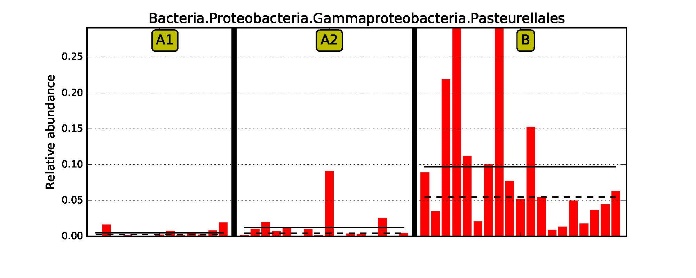


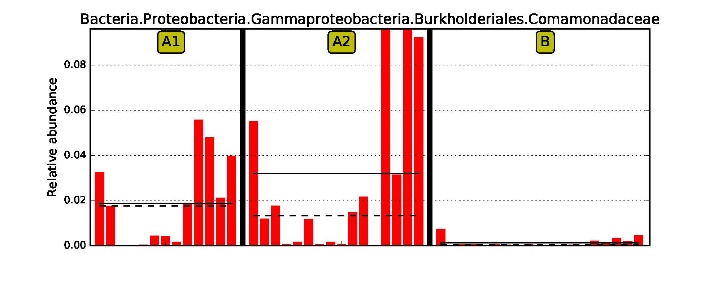

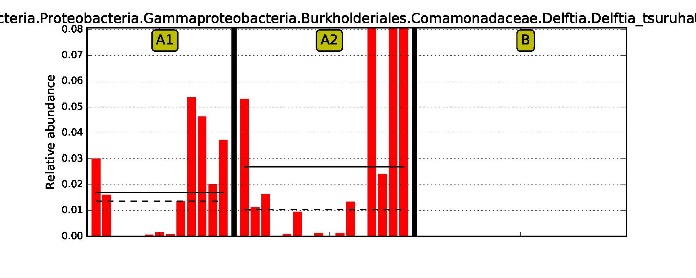

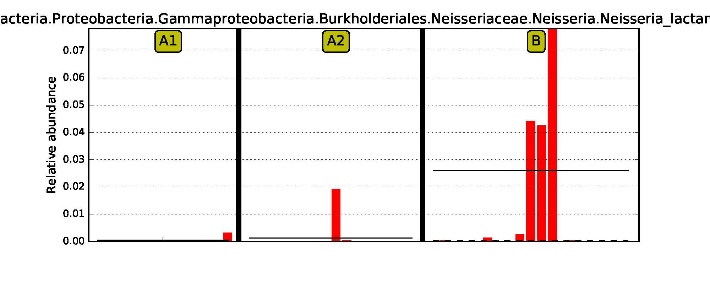


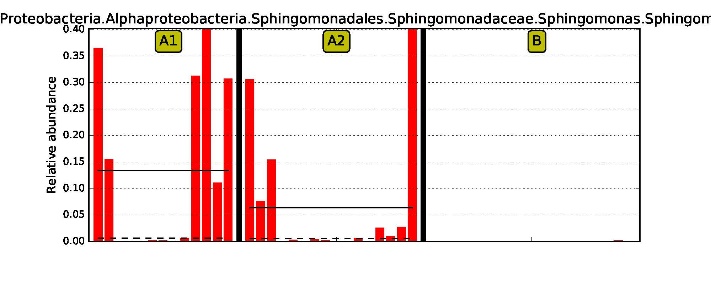

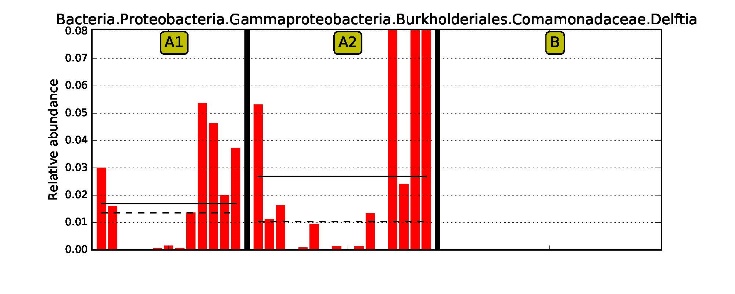

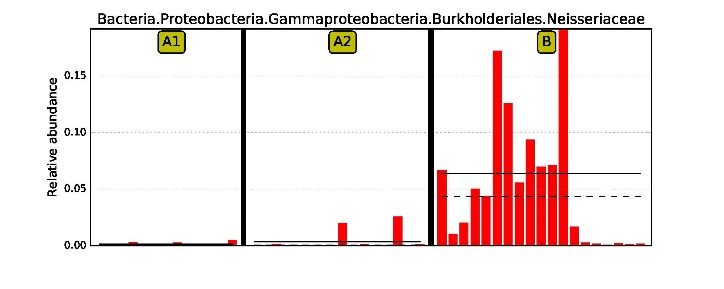


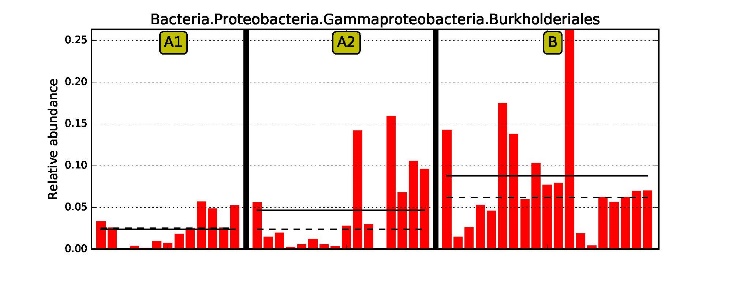


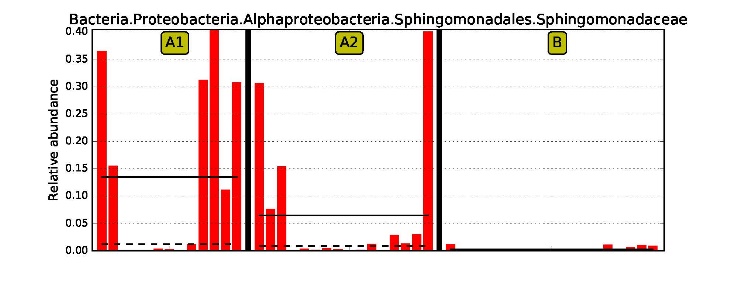

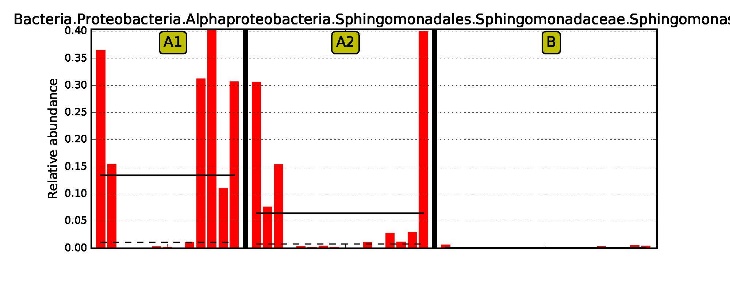


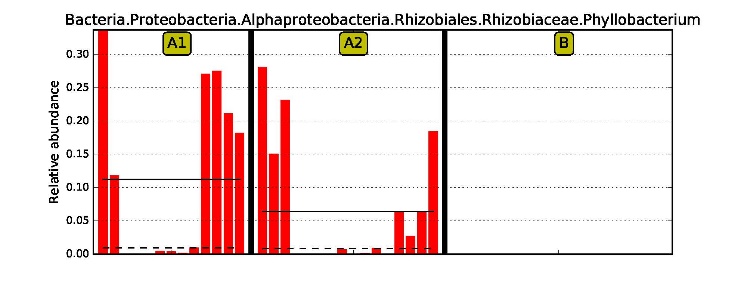


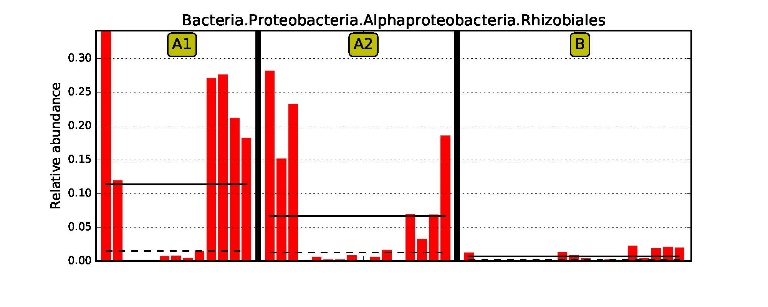

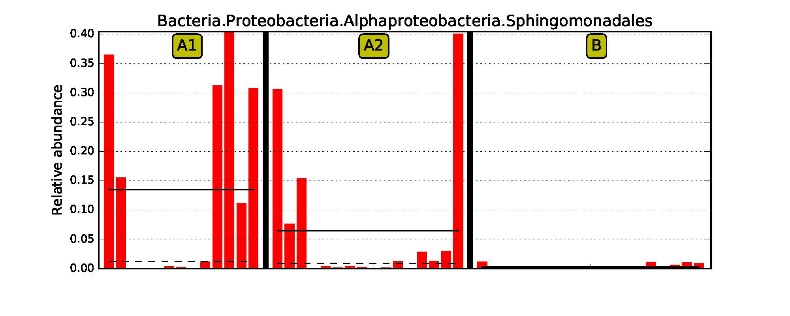


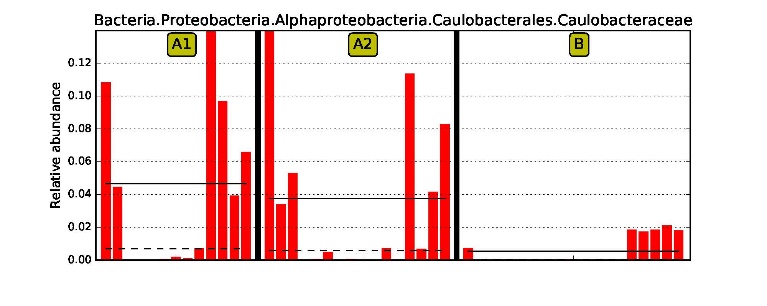

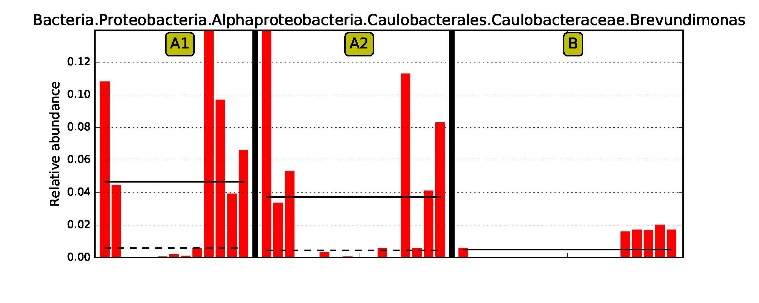

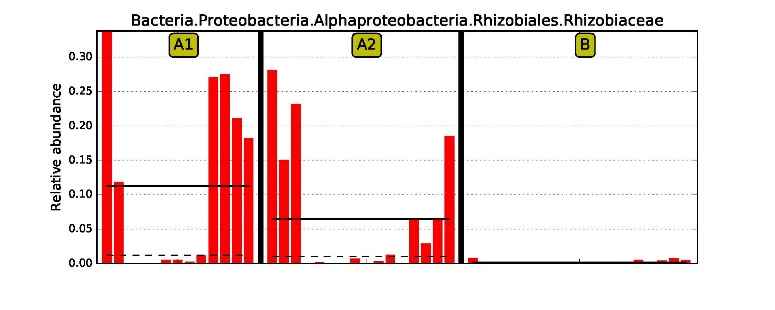


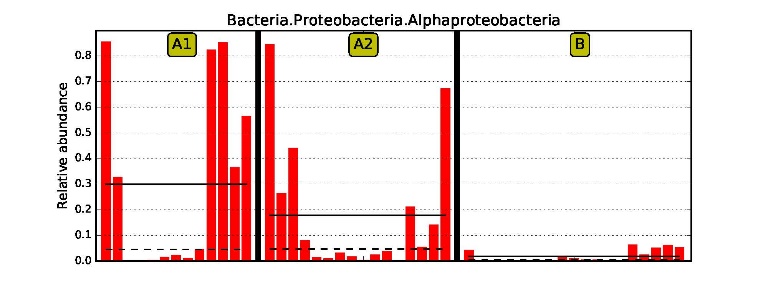

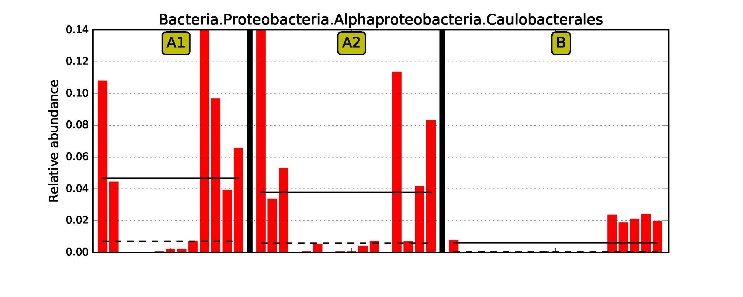


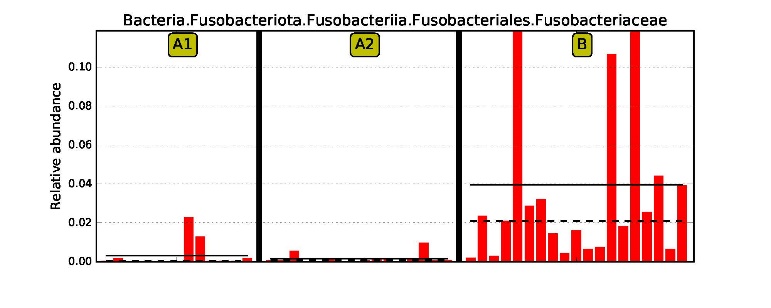

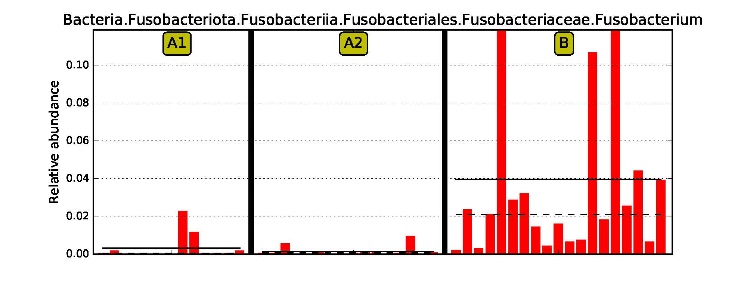


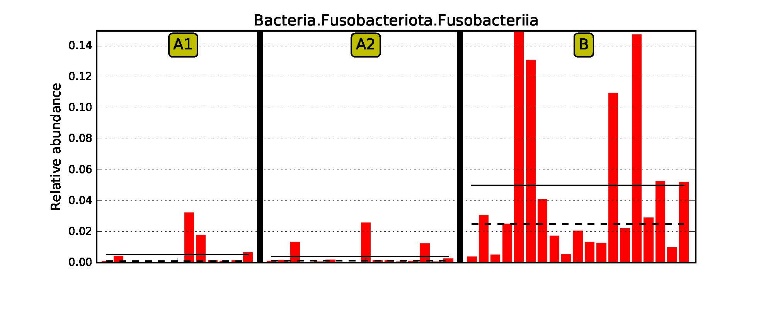


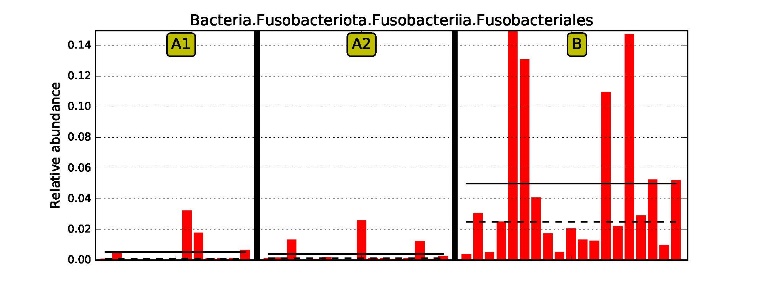


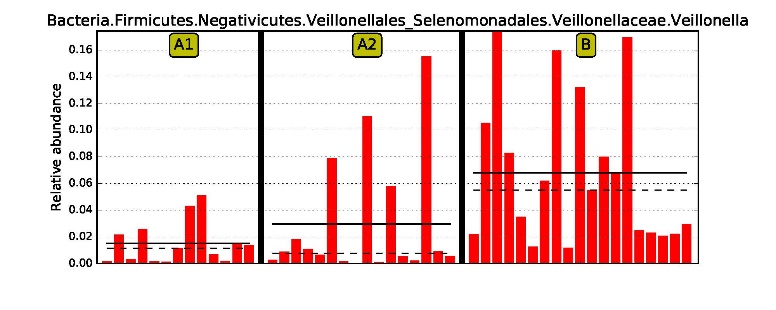


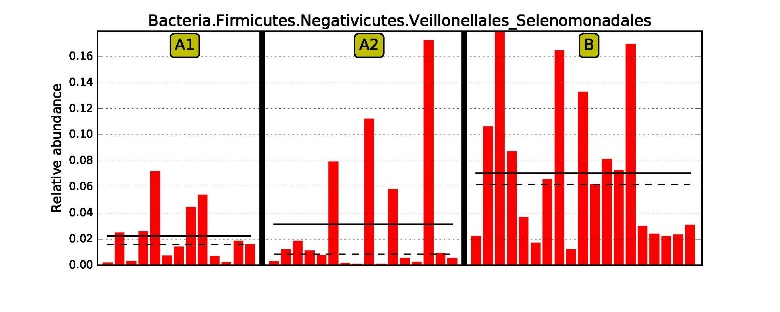

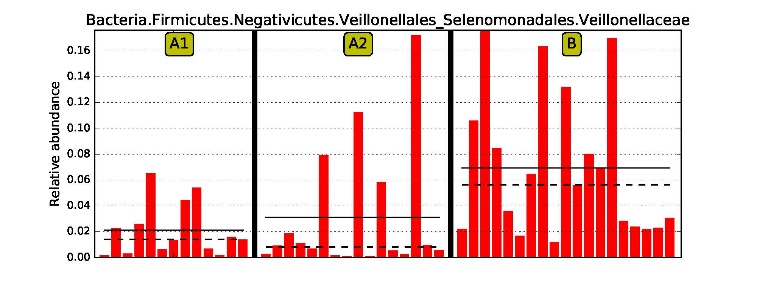

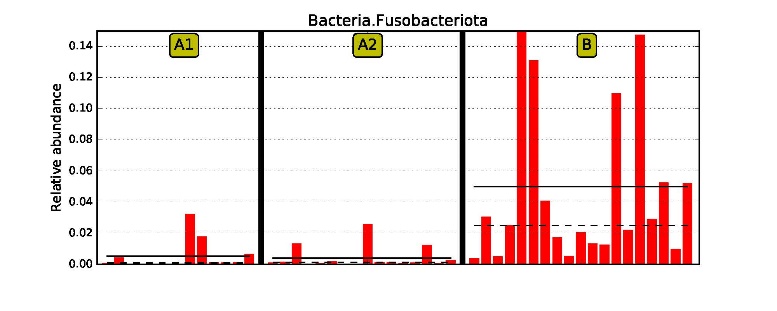


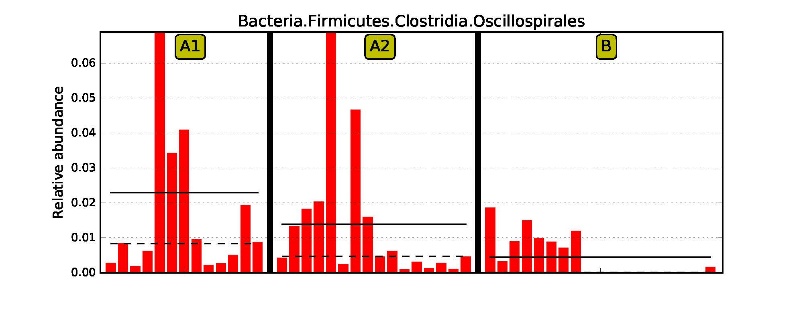

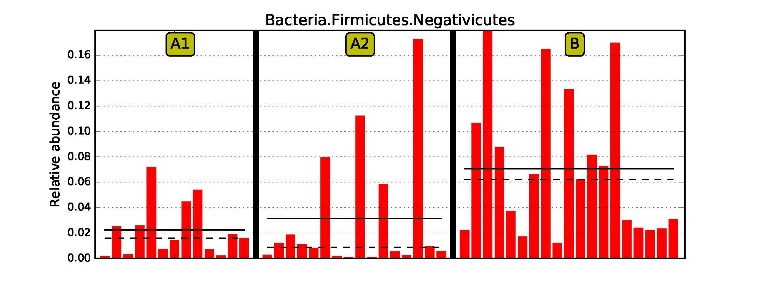


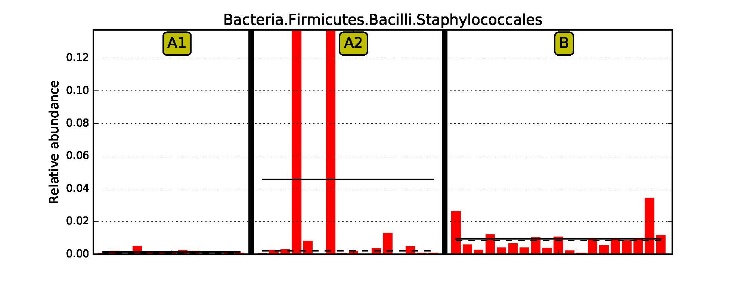


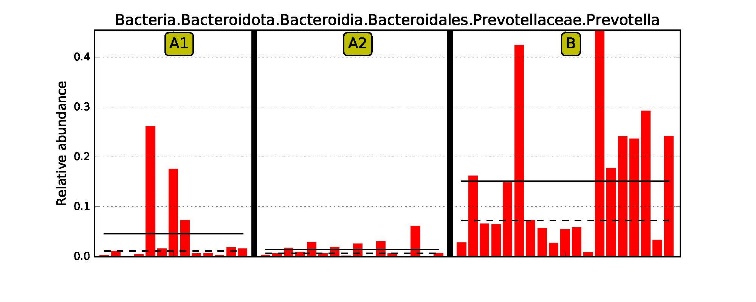

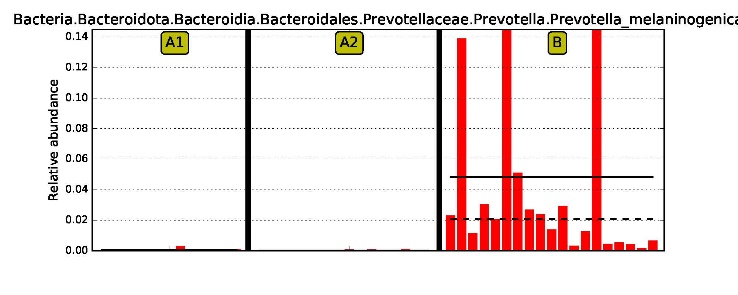

Supplement: Supplementary file 1 — Additional file 1: Table 1. Alpha diversity indices of all oropharyngeal samples. Fig. 1. Multiple rarefaction curves of the richness and Rank Abundance. Fig. 2 NMDS based on the Bray-Curtis distances at the OTU level at 97% identity. Fig. 3 All significantly different phyla and genera detected by LefSe. [file 13052_2022_1279_MOESM1_ESM.docx]
